# Effect of multidisciplinary intensive targeted care in improving diabetes mellitus outcomes: a randomized controlled pilot study – the Integrated Diabetes Education, Awareness and Lifestyle modification in Singapore (IDEALS) Program

**DOI:** 10.1186/s13063-019-3601-3

**Published:** 2019-09-02

**Authors:** Eberta Tan, Joan Khoo, Linsey Utami Gani, Roy Debajyoti Malakar, Tunn Lin Tay, Prasanna Sivanath Tirukonda, Jia Wen Kam, Aung Soe Tin, Tjun Yip Tang

**Affiliations:** 1 Department of Endocrinology, Changi General Hospital, Singapore, Singapore; 2 Department of Renal Medicine, Changi General Hospital, Singapore, Singapore; 3 Department of Interventional Radiology, Changi General Hospital, Singapore, Singapore; 4 Clinical Trials and Research Unit, Changi General Hospital, Singapore, Singapore; 5 Health Services Research Department, Changi General Hospital, Singapore, Singapore; 6 Department of Vascular Surgery, Singapore General Hospital, Singapore, Singapore

**Keywords:** Diabetes mellitus, Multidisciplinary care, Patient empowerment, Microvascular complications, Macrovascular complications, Atherosclerosis

## Abstract

**Background:**

There is a global pandemic of type 2 diabetes mellitus (T2DM), especially in Asia. Singapore has a prevalence of T2DM at 10.5%, which is higher than the world average of 8.8%. Multiple studies have shown that multidisciplinary, team-based, coordinated care has been associated with improved measures of quality care and reduced healthcare utilization. Patients with poor glycemic control and nephropathy are at the highest risk of developing cardiovascular complications and renal failure. In this study, we aimed to investigate the impact of intensive multidisciplinary diabetes mellitus care with patient empowerment versus routine clinical care on the rate of progression of micro and macrovascular complications and peripheral atherosclerotic burden, as measured by changes in femoral intima-media thickness (IMT) in patients with persistently elevated HbA1c and nephropathy.

**Methods:**

The study is a single-center randomized controlled trial (RCT) with two study arms - intensive diabetes mellitus care versus routine clinical care. Patients in the intensive arm will receive care from a multidisciplinary team consisting of an endocrinologist, diabetes nurse educator, dietitian, renal pharmacist and medical social worker for counselling. In addition, patients will be provided with tools for self-care empowerment such as glucometers, blood pressure monitors and android tablets to facilitate care, monitoring and education. Patients in the routine clinical care arm will receive standard clinical care. Follow up (FU) will be for 3 years. Primary outcomes include cardiovascular events, rate of progression of nephropathy and development of end-stage renal disease. Secondary endpoints include the proportions of patients with documented improved control of cardiovascular risk factors (HbA1c, blood pressure, low density lipoprotein-C (LDL-C), reduction in body weight), frequency of hypoglycemia, hospitalization days and changes in femoral IMT. We will also examine the prevalence of peripheral atherosclerosis and the predictive value and usability of lower extremity arterial ultrasound to predict cardio-cerebrovascular events, amputation and peripheral intervention.

**Discussion:**

Diabetes mellitus carries significant healthcare costs. Patients with poor glycemic control and nephropathy are at highest risk of developing cardiovascular complications and renal failure. Intensive diabetes mellitus care with patient empowerment may lead to sustained glycemic control, reduction of clinical complications and progression of nephropathy, and incidence of cardiovascular complications.

**Trial registration:**

ClinicalTrials.gov, NCT03413215. Registered on 29 January 2019.

**Electronic supplementary material:**

The online version of this article (10.1186/s13063-019-3601-3) contains supplementary material, which is available to authorized users.

## Introduction

### Background

The number of individuals with type 2 diabetes mellitus (T2DM) in Singapore, a Southeast Asian city-state with a population of 6.5 million, is estimated to grow from 400,000 to 670,000 by 2030 and to an alarming 1.0 million by 2050 with the continuing rise in the prevalence of obesity [1]. Total economic costs per working-age patient with T2DM in 2010 were estimated to be US$5,646, with total economic costs of US$787 million; this is estimated to rise to US$7,791 per patient and total economic costs of US$1,867 million by 2050 [2]. The presence of complications and need for inpatient care are major contributors to these increasing costs. Diabetic nephropathy is a major cause of morbidity and mortality. Asian ethnicity, uncontrolled hypertension and proteinuria are major risk factors for progression to end-stage renal failure [3]. Control of multiple risk factors, as in the Steno-2 study [4], has been shown to improve cardiovascular outcomes, increase the length of time free from incident cardiovascular disease, reduce the risk of death from all causes and reduce the progression of nephropathy as measured by worsening proteinuria, glomerular filtration rate (GFR) and serum creatinine. Previous trials have also suggested the utility of the chronic care model [5], structured recall [6], regular nurse contact [7], technology-enabled diabetes self-management education and support [8, 9] and a multifaceted approach in dealing with diabetes mellitus [6]. While most researchers have agreed that good glycemic outcomes are achievable through committing to appropriate intensive therapy and a team approach [10], there are multiple barriers to the implementation of quality diabetes mellitus care. In particular, barriers to good diabetes mellitus care can include the absence of a system to prioritize clinical resources for patients who require more intensive management, inadequate self-empowerment of patients, inadequate use of effective behavioral modification techniques, ineffective use of technology to enable diabetes self-management education and support and the need to self-fund glucose monitoring devices and monitoring and treatment consumables.

Accordingly, we aim to find out whether stratifying patients according to their risk of developing diabetic complications, and channeling purposefully structured clinical resources to high-risk patients will be more effective than usual care in controlling diabetes mellitus and cardiovascular risk factors and in reducing clinical event rates. We plan to structure clinical resources such that these patients will have accessibility to interdisciplinary team clinic consultations, interspersed with remote follow up of their conditions by diabetes nurse educators (DNEs). The use of messaging systems, a diabetes-specific smartphone application and smart tablets with diabetes self-management educational material will be individualized for patients, according to their preferences for communication and their technological literacy. The aim is to test whether these changes to the diabetes healthcare system will improve outcomes, yet remain sustainable in the current climate of heavy clinical workloads.

Diabetic macrovascular disease, manifested by accelerated atherosclerosis, and its complications are the leading causes of morbidity and mortality in patients with T2DM. Individuals with diabetes mellitus have twofold to fourfold higher peripheral rates of arterial disease and an approximately 15 times greater rate of lower extremity amputations [11]. At present, atherosclerotic lesions in the carotid arteries detected by ultrasound appear to represent total atherosclerotic burden [12] but this has been found to be only weakly related to plaque burden in other peripheral arteries [13]. Compared with carotid arterial plaques, the association between lower extremity atherosclerosis and general atherosclerosis has received much less attention. Our research aims to measure changes in femoral intima-media thickness (IMT) in high-risk patients with diabetes mellitus in response to intensive intervention, and investigate whether imaging to determine peripheral atheroma burden can help in predicting and preventing urgent and emergent peripheral vascular disease intervention.

## Methods

### Trial design

This is a single-center, randomized controlled trial of intensive diabetes mellitus care with patient empowerment versus routine clinical care.

### Aims and hypotheses

#### Main hypothesis

Intensive diabetes mellitus care involving the following strategies will be more effective than usual care in controlling diabetes mellitus, controlling multiple cardiovascular risk factors, slowing down the progression of nephropathy and peripheral atherosclerosis, reducing clinical event rates and decreasing the visits to specialist clinics and admissions to hospital for diabetes-related complications:

1. Channelling patients at high risk of developing diabetic complications to interdisciplinary team clinic consultations
2. Active counselling and teaching of self-care skills by allied health professionals (DNEs, medical social workers (MSWs), pharmacists)
3. Closer, remote follow-up via telephone aided by simple technology to facilitate diabetes self-management education and support by the DNE and MSW between clinic visits

#### Secondary hypotheses and objectives

We will be examining the prevalence of peripheral atherosclerosis and the value of lower extremity arterial ultrasound in predicting cardio-cerebrovascular events and/or amputation, and in reducing the risk of hospital admissions for critical limb ischemia.

### Study population

Patients seen by endocrinologists for T2DM management at a regional hospital diabetes outpatient clinic in Singapore will be screened for eligibility for the study.

### Eligibility criteria

Patients will be considered eligible for the study if they fulfil the following criteria:

1. Aged 21–70 years
2. Poorly controlled T2DM as defined by HbA1c ≥ 9%
3. Nephropathy as defined by estimated GFR (eGFR) 30–60 ml/min and/or proteinuria classified as urine protein > 0.5 g/day and/or urine albumin:creatinine ratio (ACR) >30 mg/mmol on two consecutive measurements 3 months apart

Patients will be excluded from study participation if any of the following criteria are present:

1. Type 1 diabetes mellitus defined as a history of ketosis at diagnosis (acute symptoms with heavy ketonuria (urine value > 3+) or ketoacidosis) or continuous requirement of insulin within 1 year of diagnosis.
2. Psychiatric conditions being treated by medications that adversely affect patients’ weight and stability of their mental health.
3. Patients on weight loss medications or have had bariatric surgery, which may affect their mental health and compliance with dietary advice.
4. Life expectancy < 12 months due to advanced cancer or other life-threatening conditions, such that they will not be able to perform self-care or complete 3 years of follow-up.
5. Pregnant or lactating patients.

Patients will be withdrawn from the study if any of the following criteria apply:

1. The patient withdraws consent
2. The patient is no longer able to participate or the investigator, in discussion with the patient’s attending physician(s), decides to terminate participation on medical grounds
3. The patient becomes pregnant during the study

There will be no participant replacement in the study if a participant withdraws.

### Randomization

There will be a total of 50 patients recruited, with 25 patients randomized to the intensive arm and 25 patients to the standard care arm. Randomization will be performed by a statistician using computer-generated random number sequence with an allocation ratio of 1:1 for each group.

### Allocation

Computer generated codes numbered 1–50 will be used to assign patients to either the intensive arm or the standard care arm. The randomization procedures are as follows:

1. The physician or nurse will explain to eligible patients about the international recommendation on performing regular follow-up to detect silent risk factors and complications
2. They will also explain to patients the need to assess the effectiveness of using a team to implement structured care by comparing the latter with usual care
3. After the patient has signed the consent form, a study team member will open the envelope containing the randomization group and sign and date the envelope
4. The signed envelope will be kept by the nurse in a secure place for audit purposes
5. The nurse will explain to the patient whether they have been randomized to the intensive arm or standard care arm and the assessment will proceed accordingly
6. The nurse will keep a log of randomized patients and record status including study completion, premature discontinuation, withdrawal of consent or loss to follow-up

### Interventions

#### Intensive care group visits and counseling

##### Multi-disciplinary team trained in motivational interviewing techniques

Patients will be managed by a multi-disciplinary team consisting of an endocrinologist, a DNE, a renal pharmacist and an MSW. This shifts the traditional physician-centric management to structured physician, DNE, MSW and pharmacist clinic and telemedical follow-up. This multi-disciplinary team will aim to treat to treatment targets and provide comprehensive, structured, piece-meal, interactive-style counseling incorporating structured counseling on the essential self-care behavior in people with diabetes mellitus that predict good outcomes. All healthcare providers involved in this multi-disciplinary team will be trained in motivational interviewing techniques.

##### Endocrinologist clinic review

The endocrinologist clinic review will include review of blood and urine test results, weight and blood pressure. Medication will be adjusted to achieve HbA1c, low density lipoprotein-C (LDL-C) and blood pressure and urine ACR targets.

##### DNE clinic review and telephone reviews

The DNE will counsel the patient at recruitment and follow up the patient’s self-blood glucose monitoring (SBGM) readings and food diary via telephone calls and messages, and in suitable patients who prefer to use a smartphone application, via the “CGH Diabetes Diary” application. Additional face-to-face clinics sessions will be arranged according to clinical discretion. The DNE will prepare a clinic folder for each patient and create a system to facilitate appointment booking and tracking of default. Between each follow-up visit, the DNE will contact the patient by phone or email to remind them of the appointments (e.g. medical follow-up visit or laboratory tests), remind them to adhere to medications and healthy lifestyles, perform and report self- glucose monitoring, advise on insulin dose titration and provide psychosocial support, as appropriate. Before each follow-up visit, the nurse will ensure that results of all blood and urine tests ordered by the physician are available in the clinic review folder for decision making. At each follow-up visit, the patients will first see the nurse to record blood pressure, body weight and urine and blood tests in the clinic review folder. The patient’s compliance will also be checked at each visit. The patients will see the nurse after the folow-up visit, to clarify any issues and concerns. The nurse will reinforce compliance and record any changes in medications, and note any procedures or tests ordered by the doctor. After each follow-up visit, the nurse will issue summary reports to be given to patients and physicians to promote sharing of information.

##### Renal pharmacists

Renal pharmacists will be involved in the care of the patient if blood pressure measured during the endocrinologist consultation is not at target and requires more intensive medication titration. The first appointment with the pharmacist will be scheduled 2–4 weeks after the endocrinologist’s referral, during which there will be a review of the patient’s compliance to antihypertensive agents, any side effects, renal panel results if an angiotension converting enzyme (ACE)-inhibitor or angiotensin-receptor blocker (ARB) was started or increased in dose, and home blood pressure measurements. The pharmacist will measure and document the blood pressure and heart rate in clinic and adjust the dose of antihypertensive agent(s) to meet the blood pressure goal according to the hospital’s hypertension guideline. The pharmacist will also counsel and educate the patient on blood pressure management and drug therapy where appropriate. The next appointment with the patient and any necessary blood test will then be arranged; intervals between each appointment may range between 2 weeks and 3 months during the period of dose titration. Once target blood pressure has been reached and blood pressure is stable, no further follow-up with the pharmacist will be scheduled unless requested by the endocrinologist.

##### Dietitian

The dietitian will counsel the patient at recruitment and further follow-up clinic sessions will be arranged according to clinical need and discretion. Patients will be counseled on eating behavior in line with American Diabetes Association nutrition therapy recommendations for the management of adults with diabetes mellitus [14].

##### Medical social worker

The MSW will hold six follow-up sessions (three face-to-face and three telephone follow-ups), at 3-weekly intervals, with intervals for follow-up varying according to the participant’s readiness and motivation. The first session will include administration of the Diabetes Distress Scale-17 (DDS17) [15], Medication Adherence Report Scale-5 (MARS-5) [16] assessment of the patient’s knowledge of his/her condition, his/her awareness and motivation level, engagement of the caregiver (if applicable) and goal setting. The six sessions will focus on using health change methodology to discuss seven essential self-care behaviors that predict good outcome in diabetes mellitus: healthy eating, being physically active, monitoring of blood glucose, compliance with medications, good problem-solving skills, healthy coping skills and risk-reduction behavior.

##### Equipment, technology and educational material

Equipment will be provided to suitable patients when clinically indicated and these include blood pressure monitors, glucometers, glucose test strips, lancets and android tablets. Patients whose blood pressure readings are not at target at clinic appointments will be taught and encouraged to take measure their own blood pressure and provided with a blood pressure machine. Patients with HbA1c not at target or with significant risks of hypoglycemia will also be provided with a glucometer, glucose tests strips and lancets and taught how and when to perform SBGM, instructed how to use either a written or smartphone logbook, instructed to take photographs of their meals and how to communicate with DNEs via the smartphone application or via messaging systems. Patients will also be taught how to activate smartphone applications or pedometers to measure daily activity. These aim to improve and increase patient-generated health data, communication of capillary blood glucose readings between patient and healthcare professional, diet and exercise, and act as communications channels through which individualized feedback can be given to the patient to specifically target glycemic control, blood pressure and exercise targets. Patients will also be provided with handouts and with tablets containing organized educational material including easy-to-understand and relevant videos and educational material to facilitate diabetes self-care.

#### Standard care group visits and counseling

Patients randomized to the standard care group will receive usual care, which consists of:

1. Scheduled, regular, 4-monthly, specialist outpatient care visits for review of blood pressure, HbA1c and other investigations, and titration of medications as per standard of care
2. Counseling with the DNE according to standard of care and usual follow-up via a phone call with the DNE initiated by the patient
3. Provision of educational materials on diabetes mellitus as per standard of care
4. Counseling with the dietitian according to standard of care, based on the American Diabetes Association nutrition therapy recommendations for the management of adults with diabetes mellitus [14]

### Outcomes

The following endpoints will be evaluated.

#### Primary endpoints

The primary outcome measures are (1) rate of deterioration of chronic kidney disease (rate of decline of eGFR or worsening of proteinuria) or development of end-stage renal disease, whichever events happen and (2) cardiovascular events (composite events of acute myocardial infarction, revascularization procedures, heart failure, unstable angina, arrhythmia, stroke, transient ischemic attacks requiring hospital admissions) and related death, whichever events happen.

#### Secondary endpoints

Secondary outcomes include (1) proportion of patients with documented improved control of risk factors and fulfilment of two or more of the “ABC” targets as defined by (a) HbA1c < 7%, (b) blood pressure < 140/90 mmHg and (c) LDL-C < 2.6 mmol/L and/or two of the following changes in risk factor control: (d) at least 0.5% reduction in HbA1c, (e) at least 5 mmHg reduction in systolic BP, (f) at least 0.5 mmol/L reduction in LDL-C, (g) at least 3% reduction in body weight, (2) frequency of hypoglycaemia (in the last 3 months) (3) number of hospitalization days and (4) changes in femoral IMT.

#### Other endpoints

Other endpoints include (1) change in the MARS-5 for compliance and (2) change in the DDS17 scores.

#### Vascular endpoints

Patients in both the intensive and standard care groups will undergo femoral vascular imaging at 0 months, 12 months and 36 months. Femoral vascular imaging software will be used to measure IMT.

The aims in studying patients’ vascular outcomes will be (1) to determine the prevalence of and risk factors for peripheral atherosclerosis indicated by the presence of lower extremity arterial atherosclerotic plaques, (2) to determine whether lower-extremity arterial ultrasound examination constitutes a more useful approach to screening Asian patients with T2DM for generalized atherosclerosis compared with the measurement of only one of these parameters, (3) to examine whether occurrence of lower extremity arterial plaques increases the incidence of cardio–cerebrovascular events and verify whether the presence of lower extremity atherosclerosis further increases the cardio-cerebrovascular risk independent of usual cardiovascular risk factors in patients with T2DM and (4) to document the distribution of peripheral vascular disease and severity of stenosis/occlusions in this population of patients and study whether this could be an early predictor of amputation/peripheral intervention.

### Participant timeline

**Fig. 1:**
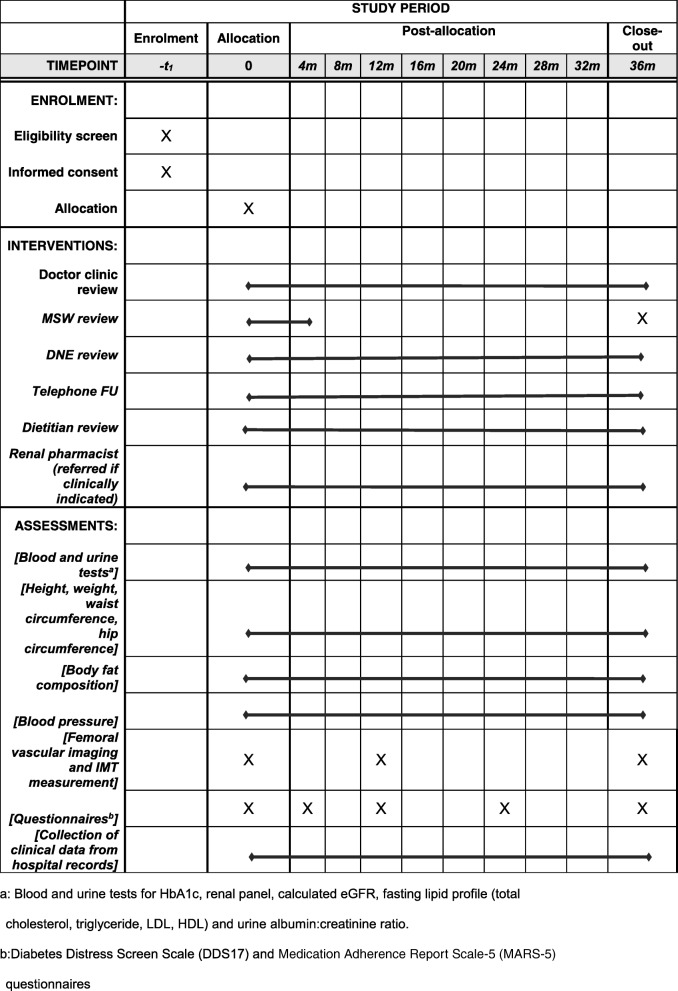
Standard protocol items: recommendation for interventional trials (SPIRIT) schedule of enrolment, interventions and assessments. ^a^Blood and urine tests for HbA1c, renal panel, calculated estimated glomerular filtration rate (eGFR), fasting lipid profile (total cholesterol, triglyceride, low-density lipoprotein cholesterol (LDL), high-density lipoprotein cholesterol (HDL)) and urine albumin:creatinine ratio. ^b^Diabetes Distress Screen Scale (DDS17) and Medication Adherence Report Scale-5 (MARS-5) questionnaires. m, month; MSW, medical social worker; DNE, diabetes nurse educator; FU, follow up; IMT, intima-media thickness

Figure 1 shows the Standard protocol items: recommendation for interventional trials (SPIRIT) schedule of enrolment, interventions, and assessments (Additional file 1).

### Sample size

The proposed sample size of 25 participants in each group is adequate to estimate the mean of the eGFR between the two groups of participants, effect size (2.5-unit difference) using the *t* test (two-tailed alpha = 0.05 with statistical power of 0.80).

### Blinding (masking)

All analyses will be completed by a statistician who is blinded to treatment allocation and unblinding will occur after the analysis is complete.

### Data collection methods

Patients will be weighed (wearing light clothing without shoes) using a calibrated electronic scale and recorded to the nearest 0.01 kg and height will be measured with a portable stadiometer to the nearest 0.1 cm (barefoot). Body fat percentage will be measured by bioimpedance electrical analysis using the Tanita TBF-300 Goal Setter body composition analyzer. Waist circumference will be measured by a non-stretch tape at the level midway between the lowest rib margin and the iliac crest and recorded to the nearest 0.1 cm, while the patient is standing. Hip circumference will be measured at the widest circumference at the level of the symphysis pubis and gluteus maximus.

Systolic and diastolic blood pressure (BP) will be measured at the left arm with patients seated and rested, with an appropriately sized cuff placed at the level of the heart using an automated sphygmomanometer.

Venous blood and urine samples will be collected after 10 h of fasting at week 0 and thereafter before every clinic review by the endocrinologist. These include samples for testing HbA1c, renal panel, calculated eGFR, fasting lipid profile (total cholesterol, triglyceride, LDL-C, HDL-C) and urine albumin:creatinine ratio.

Baseline, 4-month (after MSW intervention) and end-of-study MARS-5 and DDS17 questionnaires will be performed in both the intervention and control groups. The MARS-5 by Horne et al. has been widely used to assess patient adherence to medication in clinical research and medical practice. The DDS17 is a 17-item scale that captures four critical dimensions of distress: emotional burden, regimen distress, interpersonal distress and physician distress.

Philips Medical systems QLAB Quantification software for IMT will be used as the primary measurement tool for bilateral common femoral arteries (CFA) and superficial femoral arteries (SFA) [12, 17]. When automated measurements are not possible due to the course of the arteries or state of disease, manual measurements will be taken at the same specified anatomical landmarks.

The IMT is defined as the distance between two echogenic lines represented by the lumen-intima interface and media-adventitia interface of the arterial wall (Table 1).

**Table 1:** Measurement details of femoral vascular imaging and IMT measurement. CFA, common femoral arteries; SFA, superficial femoral arteries

Assessment modality	B-mode ultrasonography	
Equipment	Philips IU22 (L9–3 MHz Linear Transducer) or Epiq 7 (L12–3 MHz Linear Transducer)	
Examination preset	CGH vascular arterial	
Patient position	Supine, leg externally rotated, knee slightly bent	
Femoral artery	CFA	Proximal SFA
Site of IMT measurement	A straight 1-cm segment 1.5–2 cm proximal to the CFA bifurcation Far wall	A straight 1-cm segment 1.5-2 cm distal to the CFA bifurcation Far wall

### Data management

Case record forms will be stored in locked cabinets in the research office. Research data will be stored on the designated computer, which is password protected. Only the delegated study staff will have access to the research data. The study participants’ identification will be coded and their records will be available only to the investigators.

### Statistical methods

Categorical data will be presented as frequency (percentage). Numerical data will be presented as mean (standard deviation (SD)), unless specified otherwise. Differences in baseline characteristics between two groups will be examined using the chi-square test or Fisher’s exact test for categorical variables and the two-sample *t* test or Mann-Whitney U test for continuous variables.

Logistic regression of binary outcomes such as cardiovascular events will be performed to determine the association between treatment group and potential risk factors and the outcomes. Relative risk (RR) (with 95% CI) will be calculatec. For continuous outcomes such as change in the MARS-5 and DDS17, linear regression will be used. Measure of tolerance for multicollinearity will be assessed. Coefficient B (with 95% CI) will be reported. The number of occurrences of hypoglycemia will be analyzed by Poisson regression. Measure of deviance will be checked and relative risk (RR) (with 95% CI) will be calculated.

*P* values < 0.05 will be deemed statistically significant. Statistical analysis will be performed using SPSS statistical software, version 19.0 (IBM Corp. Armonk, NY, USA). This RCT will be analyzed by the principle of intention to treat (ITT) and the per protocol (PP) approach.

### Data monitoring

There is no data safety monitoring board for this study. the principal investigator (PI) and co-investigators will perform data and safety monitoring. The PI will regularly monitor the study to verify the authenticity, accuracy and completeness of data, that the safety and rights of participants are protected and that the study is conducted in accordance with the latest approved protocol, International Conference on Harmonisation Good Clinical Practice (ICH-GCP) and all applicable regulatory requirements.

### Harms

Clinical observation and laboratory results will be used to evaluate the wellbeing of the patient on a continual basis throughout the duration of the study. In the event that the Investigator considers that the wellbeing of an individual patient is unacceptably at risk as a result of participation in the study then the individual patient will be withdrawn from the trial. In the event that the withdrawal of an individual also has wider implications for the wellbeing of other participating patients, then the study will be suspended and an investigation conducted to determine whether the trial should be continued as proposed, in a modified form or terminated.

### Collecting, recording and reporting of serious adverse events (SAEs)

A serious adverse event (SAE) is any untoward medical occurrence that:

- Results in death.
- Is life-threatening (immediate risk of death)
- Requires inpatient hospitalization or prolongation of existing hospitalization
- Results in persistent or significant disability/incapacity
- Results in congenital anomaly/birth defect
- Is a medically important event

All SAEs will be collected, documented, assessed and reported by the PI to SingHealth Centralized Institutional Review Board (CIRB) according to its guideline in the prescribed SAE form.

Complaints will be recorded in the case notes, and possible adverse events will be recorded in a separate section in the case records form. The PI and co-investigators will determine the severity, causality and expectedness of adverse events. If the investigator determines that the event may jeopardize the participant and/or may require intervention to prevent one of the other adverse event outcomes, the important medical event will be reported as serious. Causality will be defined as related/not related, and expectedness will be based on reported adverse events in the treatment.

## Ethics and dissemination

### Consent

The investigator will explain the study in full to the patient according to the approved informed consent form. The investigator will comply with the ICH-GCP Guidelines and to the ethical principles that have their origin in the Declaration of Helsinki. Informed consent will not be taken by the primary physicians, but explained by another study team member who is not the participant’s primary physician.

Participants will be reassured that they are not obliged to participate in the research, and that declining to participate in the study or choosing to withdraw from the study will not result in prejudicial treatment, resentment or abandonment by the primary physician. A copy of the signed informed consent form will be provided to the patient for his/her retention and reference.

### Confidentiality of data and patient records

The medical records of all participants that are collected for study purposes will only be accessible to investigators. Participants will be given study numbers and these will not be identifiable to non-investigators. The study investigations will be performed in individual rooms.

### Access to data

The PI and co-investigators will have access to the final trial dataset.

## Discussion

Multifactorial interventions of measuring blood glucose, blood pressure and LDL-C have been demonstrated to decrease cardiovascular complications and mortality in type 2 diabetes mellitus [4, 18]. Nurse-managed diabetes mellitus outpatient-management protocols [6], self-monitoring of blood glucose and training in medication adjustment [7] and diabetes self-management education programs are effective in increasing patient knowledge, skills and motivation for disease management with associated improvements in outcomes [19].

With increasing healthcare costs and incidence of diabetes mellitus in Asian countries such as Singapore [2] and the rising availability and use of the Internet, smartphones and social media, facilitating self-monitoring and remote follow-up using technology in patients at high risk of developing diabetic complications is likely to be an increasingly attractive and cost-effective care model compared to frequent hospital visits with healthcare provider-centric treatment. Mobile phones are now omnipresent with worldwide usage rates nearing 100% (96% globally, 128% in developed countries and 89% in developing countries). Singapore has a smartphone user rate of 91% [20, 21]. Technology-enabled diabetes self-management solutions have been shown to significantly improve HbA1c, with the most effective interventions incorporating all the components of a technology-enabled self-management feedback loop that connects people with diabetes mellitus and their healthcare team using two-way communication, analysis of patient-generated health data, tailored education and individualized feedback [8]. On average, mobile phone-based interventions with clinical feedback lead to improvements in HbA1c compared to standard care or other non-mHealth approaches by as much as 0.8% in patients with T2DM, at least in the short term (≤ 12 months) [22].

However, not all digital interventions have been successful, due to psychosocial determinants such as psychological distress and inadequate social support, which influence diabetes mellitus self-care [23], resistance or variable competence and comfort with using technology for self-care and contact with healthcare providers [24], possibly secondary to lower literacy levels or advanced age with poorer vision and dexterity, difficulty for the individual to synthesize and understand how health data relate to their health and behavior and need for supervision, affirmation and sometimes face-to-face interactions from their healthcare provider for behavioral changes [25] and gradual loss of interest as the novelty of the intervention wears off [26]. It is known that certain types of educational methods and media work better than others in individuals with low health literacy [27–30] and that mobile health may not be appealing and effective in all individuals. As such, our intervention program incorporates assessment of health literacy and counseling of patients by medical social workers to decrease distress, improve support and self-care, and training of physicians, DNEs and dietitians in motivational interviewing and solution-focused coaching techniques*.* Educational materials provided and educational and communication methods utilized for each patient will also be customized based on the healthcare team’s assessment of the patient’s suitability or preference (face-to-face sessions, use of technology for communication, written handouts, electronic information articles, videos, etc.). Knowledge gaps that are identified will guide the focus of education.

This study will serve as a platform for developing a patient-centric program, integrating self-care and holistic input from allied health workers, aiming to optimize resource allocation to improve patient outcomes while remaining sustainable in the current climate of heavy clinical workloads.

## Additional file

Additional file 1: SPIRIT 2013 checklist: recommended items to address in a clinical trial protocol and related documents. (DOC 123 kb)

## Data Availability

Findings will be disseminated in peer-reviewed scientific journals and presented at scientific meetings and conferences. The datasets generated and/or analyzed during the study will be available from the corresponding author on reasonable request.

## Abbreviations

ACE-inhibitor: Angiotension converting enzyme inhibitor
ARB: Angiotensin-receptor blocker
CFA: Common femoral arteries
CIRB: Centralized Institutional Review Board
DDS17: Diabetes Distress Scale-17
DNE: Diabetes nurse educator
eGFR: Estimated glomerular filtration rate
GFR: Glomerular filtration rate
HbA1c: Glycated hemoglobin
ICH-GCP: International Conference on Harmonisation Good Clinical Practice
IMT: Intima-media thickness
ITT: Intention to treat
LDL-C: Low density lipoprotein-C
MARS-5: Medication Adherence Report Scale-5
MSW: Medial social worker
OR: Odds ratio
PI: Principal Investigator
PP: Per protocol
RCT: Randomized controlled trial
SAE: Serious adverse event
SBGM: Self blood glucose monitoring
SD: Standard deviation
SFA: Superficial femoral arteries
T2DM: Type 2 diabetes mellitus
urine ACR: Urine albumin:creatinine ratio
